# Population wide testing pooling strategy for SARS-CoV-2 detection using saliva

**DOI:** 10.1371/journal.pone.0263033

**Published:** 2022-01-28

**Authors:** Eduardo Esteves, Ana Karina Mendes, Marlene Barros, Cátia Figueiredo, Joana Andrade, Joana Capelo, António Novais, Carla Rebelo, Rita Soares, Ana Nunes, André Ferreira, Joana Lemos, Ana Sofia Duarte, Raquel M. Silva, Liliana Inácio Bernardino, Maria José Correia, Ana Cristina Esteves, Nuno Rosa

**Affiliations:** 1 Universidade Católica Portuguesa, Faculty of Dental Medicine (FMD), Center for Interdisciplinary Research in Health (CIIS), Viseu, Portugal; 2 Faculty of Health Sciences, University of Beira Interior, Covilhã, Portugal; 3 Centro Hospitalar Tondela Viseu, Viseu, Portugal; 4 Department of Biology, CESAM, University of Aveiro, Aveiro, Portugal; "INSERM", FRANCE

## Abstract

SARS-CoV-2 pandemic has forced frequent testing of populations. It is necessary to identify the most cost-effective strategies for the detection of COVID-19 outbreaks. Nasopharyngeal samples have been used for SARS-CoV-2 detection but require a healthcare professional to collect the sample and cause discomfort and pain to the individual. Saliva has been suggested as an appropriate fluid for the diagnosis of COVID-19. We have investigated the possibility of using pools of saliva samples to detect SARS-CoV-2 in symptomatic and asymptomatic patients. Two hundred and seventy-nine saliva samples were analyzed through RT-PCR of Envelope, Nucleocapsid and Open Reading Frame 1ab genes. Reproducibility assays showed an almost perfect agreement as well as high sensitivity (96.6%), specificity (96.8%), positive predicted value (96.6%), and negative predicted value (96.8%). The average Cycle Threshold of the genes detected was 29.7. No significant differences (*p* > 0.05) were detected when comparing the cycle threshold average of two consecutive reactions on the same positive saliva samples. Saliva samples have a higher median viral load (32.6) than in nasopharyngeal samples (28.9), although no significant differences were detected (*p* > 0.05). Saliva-pool samples allowed effective SARS-CoV-2 screening, with a higher sensibility (96.9%) on 10-sample pools than in 20-sample pools (87.5%). Regardless of pools size specificity was high (99.9%) and an almost perfect agreement was observed. Our strategy was successfully applied in population wide testing of more than 2000 individuals, showing that it is possible to use pooled saliva as diagnostic fluid for SARS-CoV-2 infection.

## Introduction

The COVID-19 pandemic, caused by SARS-CoV-2, has created challenges at a global scale [1–11]. Currently, the standard diagnostic method for COVID-19 is to perform a real-time polymerase chain reaction (RT-PCR) of a sample taken by smear (swab) from the naso and oropharynx. The replacement of the nasopharynx swabs with less invasive collection techniques is desirable and has been moderately explored [8, 12–18]. Saliva is commonly used to diagnose viruses similar to SARS-CoV-2 and has been suggested as appropriate for the diagnosis of COVID-19 [6, 12, 15, 17, 19–21] but the detection method still needs improvement. Until now, saliva testing was less sensitive than nasopharyngeal swab [22], however recent studies have reported similar results for saliva and nasopharyngeal swabs [23]. The collection of saliva is non-invasive and can be performed by the patient him/herself, reducing the risk of cross-contamination and the need of specialized healthcare workers. Saliva is thus more amenable to frequent testing since it is safer (to the patient and to the healthcare worker), cheaper and imposes less discomfort in the individual being tested [6, 19, 24].

Institutions and authorities are forced to choose carefully which are the most cost-effective strategies to avoid spread and outbreaks. One of the most effective ways to prevent virus spreading includes the routine testing of individuals using a highly sensitive method to detect asymptomatic or pauci-symptomatic and mild cases [25]. On the other hand, mass testing, using the current nasopharynx swabs/PCR tests, represents a huge economic cost that most institutions cannot support [16–19]. The burden of mass testing can be alleviated by means of pool-based strategies [26–29]. The rational is that if a pool of samples is positive then, and only then, individual samples of the pool are subsequently tested, which can markedly reduce the number of tests being carried out. But cost is not the only factor, indeed pooling or group testing of specimens is faster than individual testing and saves resources, by expanding the detection capacity while limiting the risk of reagent shortage [30], as well as, reducing the environmental impact of all the residues produced by mass testing [31, 32].

For nasopharyngeal samples it has been established that an individual positive sample can still be detected in pools of up to 32 samples, and even 64 samples, provided that additional polymerase chain reaction (PCR) amplification cycles are conducted with a sensitivity of 96% [6, 8, 22]. Currently, several studies on saliva pool-testing for SARS-CoV-2 have been conducted [13, 27–29]. However complex protocols, prone to error, are frequently followed [33] and the maximum number of samples that should be present in a pool without losing sensibility is not clearly established, although some studies report small pool samples of less than 20 samples per pool. In fact, there is no consolidated information about the use of saliva in pool testing [13, 27–29].

Saliva sample pooling has the advantages of sample pooling described above as well as the added advantage of a simpler and more comfortable collection for the patient. Children and senior patients will benefit tremendously with this strategy. Therefore, the aim of this study is to provide evidence on the use of saliva sample pools for SARS-CoV-2 detection using large saliva pools of 20 and 10 samples.

## Material and methods

### Sampling process, sample and research participants

Saliva (SAL) and Nasopharyngeal Swab (NPS) samples were collected at Centro Hospital Tondela Viseu (Viseu, Portugal), at the Portuguese Football Federation (Lisboa, Portugal), at the Municipality of Viseu and at Faculty of Dental Medicine of the Portuguese Catholic University (Viseu, Portugal).

Passive drooled saliva samples (2mL) were collected into 50mL sterile tube without stabilizers using a previous established protocol [24]. Human nasopharyngeal/oropharyngeal swab specimens were collected from the same donors, according to the Portuguese Directorate-General of Health (DGS) guidelines and placed in viral transport medium. This study used 184 saliva samples for SARS-Cov-2 detection method development (160 SARS-CoV-2 negative samples and 24 positive samples); for the pool assays, 132 samples were analysed (130 negative and 2 positive). The community test was performed with 2017 saliva samples from 216 female and 1801 male volunteers. The average of ages was 17.1 (±10.7) years and 12.9 (±8.5) year respectively (S1 Table). Saliva collections in minors was executed under the supervision of the legal tutor.

This is a cross-sectional study focused on detecting SARS-CoV-2 viral load on saliva pools for population wide testing. To achieve this goal, we studied: 1) the potential of saliva for the detection of SARS-CoV-2 using a simplified procedure; 2) the sensibility/specificity of detection method in saliva; 3) the potential of pooling saliva specimens for the detection of SARS-CoV-2.

This study was carried out in accordance with the Helsinki Declaration and the Oviedo Convention. The ethical aspects of the present study were reviewed and approved by the Ethics Committee for Health at Centro Hospitalar Tondela Viseu. Written informed consent including purpose of the study, data confidentiality, rights of participation, and the right to withdraw from the study at any time was provided by every participant before study enrolment.

### Sample storage and pre-treatment

Samples were sent to the lab on a refrigerated container to perform sample inactivation, RNA extraction and further SARS-Cov-2 detection by RT-PCR. All extracted RNA samples were stored at -80°C until analysis. All samples were destroyed upon completion of the study.

#### 1. RNA extraction

To compare paired SAL and NPS samples, total RNA was extracted from 200 μl of the NPS viral transport medium and saliva samples, using RNAdvance Viral XP kit (Beckman Coulter, Indianapolis, United States) using Biomek i5 Automated Workstation liquid handling (Beckman Coulter, Indianapolis, United States) according to manufacturer’s protocol.

To simplify procedures, reduce costs and time of analysis, we optimized a direct RT-PCR approach for SARS-Cov-2 detection combining proteinase K and heat-inactivation. For this purpose, a volume of 100 μL of sample (single or pooled) was mixed with 20 μL of Proteinase K (20 μg/mL, NZYTech, Lisboa, Portugal) followed by incubation at 57°C (15 min) and at 95°C for 15 min (enzyme and viral inactivation). This strategy was used to determine sensitivity and reproducibility for pooling assays.

#### 2. SARS-CoV-2 detection

Viral load determination was performed using reverse transcription-polymerase chain reaction (RT-PCR) analysis using the Novel Coronavirus (2019-nCOV) RT-PCR detection Kit (Shanghai Fosun Long March Medical Science CO. Ltd, China) for O (*ORFa1b*), *N* (Nucleocapsid phosphoprotein coding gene) or *E* (Envelope) gene fragments of SARS-CoV-2, according to manufacturer’s protocol. RT-PCR reactions were performed on a CFX96 Touch Real-Time PCR Detection System (Bio-Rad, California, United States). Samples were considered positive, when amplification of two or three SARS-CoV-2 gene fragments were amplified below 40 cycles (C_T_ value).

#### 3. Assay design

For sensitivity determination we spiked infectious viral particles RNA UN kit SARS-CoV-2 Reference Material Kit) into healthy donor saliva at 1:10 ratio for contrived positive sample, after SAL and NPS COVID-19 detection. For reproducibility assay we performed two separate RT-PCR reactions for the same saliva samples. For evaluation of pooling saliva samples for SARS-CoV-2 screening prior to RNA extraction, we arbitrarily chose 2 positive samples and 130 negative samples, previously determined. A total of 32 pools of 10 samples (1 positive + 9 negative) and 32 pools of 20 samples (1 positive + 19 negative) were prepared. The negative samples were mixed into pools of different sizes containing equal volumes of 10 and 20 unique samples, 32 pools each. Negative pools were prepared with different samples to determine whether different negative-sample composition in the pool affected the detection of positive samples. Sensitivity and sensibility were determined as described [34–36].

#### 4. Data treatment and statistical analysis

Average cycle threshold (C_T_) was calculated using Microsoft Office Excel 365 software (Microsoft, Redmond, WA). Kappa (Ƙ) statistics value, sensitivity, specificity, positive predictive value (PPV) and negative predictive value (NPV) at two-sided 95% confidence interval (CI) using the Clopper and Pearson method, were analysed using Online GraphPad Prism version calculator (GraphPad Software, San Diego, CA, USA).

Pair analysis (Saliva and Nasopharyngeal Swab specimens) were conducted by Wilcoxon signed rank test. Pooling strategy statistical analysis was conducted by Mann-Whitney rank test. These analyses were performed using GraphPad Prism version 8 (GraphPad Software, San Diego, CA, USA).

## Results

### Saliva assay sensitivity

To determine the sensitivity of the detection of SARS-CoV-2 in saliva samples, 99 saliva samples were tested: 50 positive samples (to which a SARS-CoV-2 RNA template was added; these will be referred to as “spiked samples” from this point forward) and 49 negative samples. 48 of the 50 spiked samples were identified as positive for SARS-CoV-2, while 2 were recorded as (false) negatives (Table 1). We observed an almost perfect agreement (Ƙ = 0.96, CI 95%: 0.90–1.00) with 96% of sensibility (95% CI: 0.86–0.99) and 100% of specificity (95% CI: 0.93–1.00). The average C_T_ of three genes were: O gene C_T_ 32.7, E gene C_T_ 33.3 and N gene C_T_ 34.0.

**Table 1:** Sensitivity and specificity of SARS-CoV-2 detection on saliva samples by RT-PCR.

Saliva samples	N	Saliva samples		Sensitivity (95% CI)	Specificity (95% CI)
		Detected	Not Detected		
Positive	50	48	2	96% (0.86–0.99)	100% (0.93–1.00)
Negative	49	0	49		

Saliva samples were divided into 2 groups: a control group and a test group in which samples were spiked with a SARS-CoV-2 RNA template. Sensitivity and specificity were determined as described [34–36]. CI–confidence interval.

### Assay reproducibility

In order to test the reproducibility of the method, we tested 60 saliva samples, divided into 2 groups (29 Sars-Cov-2 spiked and 31 Sars-Cov-2 negative). Each of these samples were tested twice (Table 2). Data shows an almost perfect agreement (Ƙ = 0.93, CI 95%: 0.84–1.00) as well as hight sensitivity (96.6%; CI 95% 0.82–0.99), specificity (96.8%; CI 95% 0.83–0.99), positive predicted value (96.6%; CI 95% 0.82–0.99), and negative predicted value (96.8%; CI 95% 0.83–0.99). A false negative and a false positive were detected on the second reaction.

**Table 2:** Determination of sensitivity and specificity of saliva testing.

Saliva samples	N	Saliva samples		Sensitivity (95% CI)	Specificity (95% CI)	PPV (95% CI)	NPV (95% CI)
		Detected	Not Detected				
Positive	29	28	1	96.6% (0.82–0.99)	96.8% (0.83–0.99)	96.6% (0.82–0.99)	96.8% (0.83–0.99)
Negative	31	1	30				

RP-PCR was used to detect SARS-COv-2 in saliva samples. Each sample was analysed twice and treated as independent samples. Sensitivity, sensibility, positive predictive value (PPV) and the negative predictive value (NPV) (with a confidence interval (CI) of 95%) were determined according to [34–36]. CI–confidence interval.

**Fig 1:**
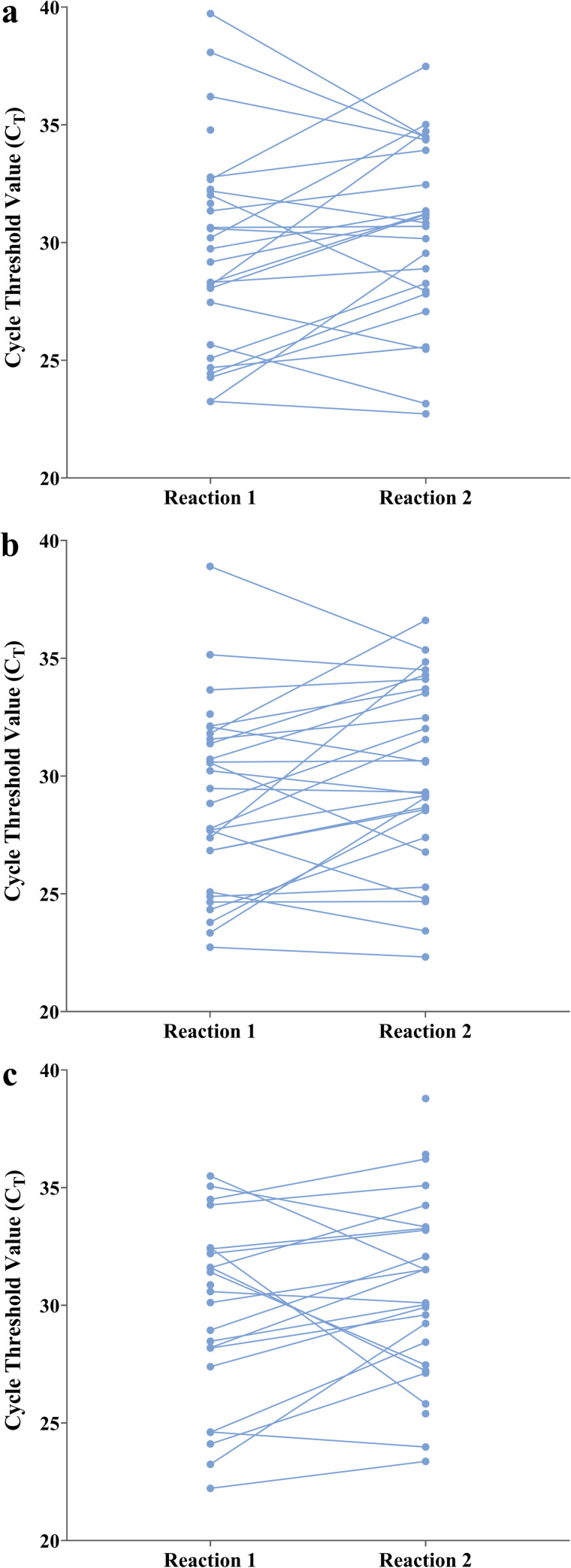
Cycle threshold (C_T_) values for SARS-CoV-2 E gene (a), N gene (b) and O gene (c) of paired saliva samples (N = 29), connected by a line, in two different RT-PCR reactions (Reaction 1 and Reaction 2), compared by Wilcoxon matched pairs signed rank test.

No significant differences (*p* > 0.05, Fig 1) were detected when comparing both reactions on positive saliva specimens, by Wilcoxon matched pair signed rank test. The average C_T_ of three genes were: O gene C_T_ 29.6; E gene C_T_ t 30.2 and N gene C_T_ 29.4.

### Comparison between saliva samples and nasopharyngeal swab samples

**Fig 2:**
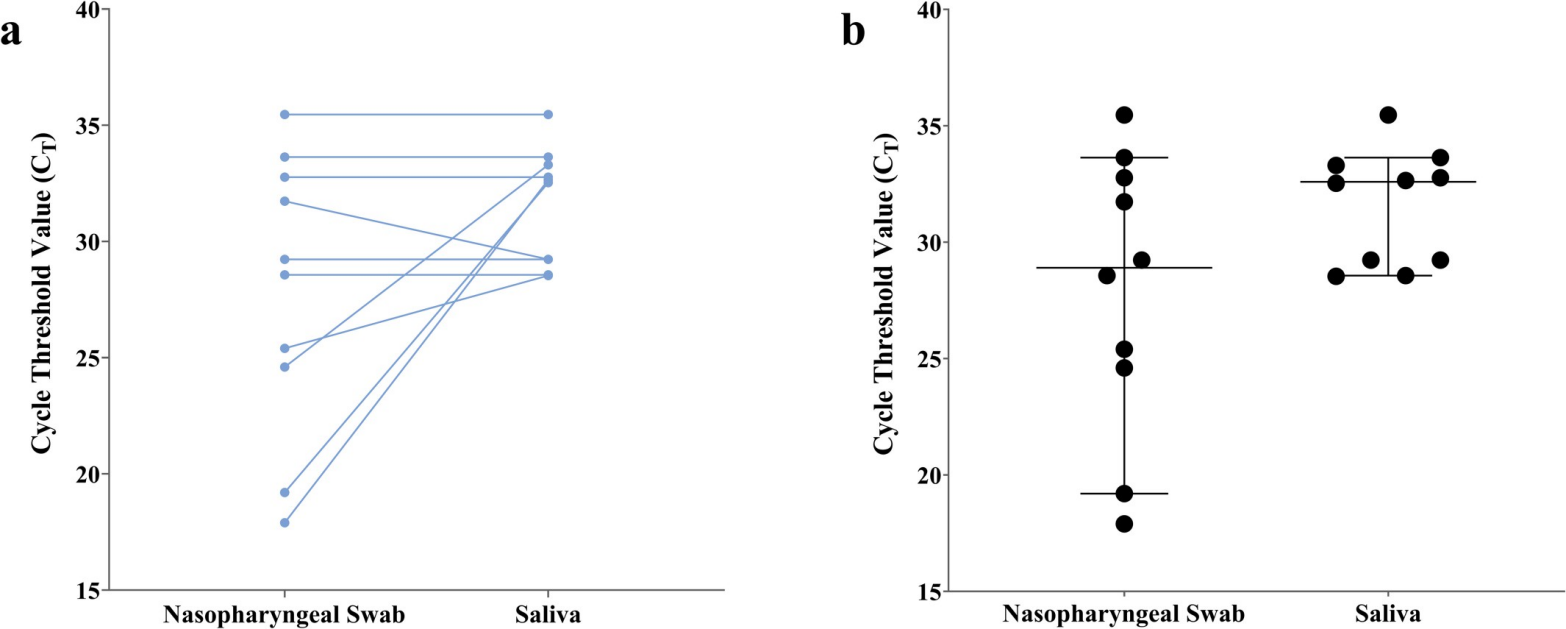
Comparison of viral loads of SARS-CoV-2 between nasopharyngeal swab and saliva specimens. a) Ct values for paired NPS and SAL samples (10 pairs). Pairs are connected by a line. b) Scatter plot with the median with 95% CI on error bars. Statistical differences were determined by Wilcoxon matched pairs signed rank test.

We evaluated the concordance between the detection of SARS-Cov-2 in saliva (SAL) and in nasopharyngeal (NPS) samples collected from the same patients (147 pairs of SAL and NPS samples, Table 3). Detection of SARS-CoV-2 on NPS samples showed a moderate agreement with SAL samples (Ƙ = 0.58, CI 95%: 0.37–0.79). We also compared the C_T_ values of the concordant positive SAL and NPS samples (10 positive pairs in NPS and SAL) by Wilcoxon matched pair signed rank test. A higher median viral load was seen for SAL (32.6) specimens compared with the median C_T_ for NPS samples (28.9) with no significant differences (*p* > 0.05, Fig 2).

**Table 3:** Determination of sensitivity and specificity in saliva (SAL) using nasopharyngeal (NPS) paired samples as gold standard.

Saliva samples	N	Nasopharyngeal Swab samples		Sensitivity (95% CI)	Specificity (95% CI)	PPV (95% CI)	NPV (95% CI)
		Detected	Not Detected				
Positive	15	10	5	66.7% (0.33–0.82)	94.7% (0.91–0.99)	62.2% (0.38–0.88)	95.6% (0.89–0.98)
Negative	132	7	125				

Sensitivity, sensibility, positive predictive value (PPV) and the negative predictive value (NPV) (with a confidence interval (CI) of 95%) were determined according to [34–36]. CI–confidence interval.

### Evaluation of the pooling saliva samples strategy for SARS-CoV-2 screening

Due to dilution pooling, it is expected a reduction on the sensitivity of SARS-CoV-2 detection by RT-PCR. To test this hypothesis, our pooling strategy was designed using 10 (Table 4) or 20 (Table 5) saliva samples per pool. Each “positive pool” contained a SARS-CoV-2 positive sample with an average of 25.6 C_T_. “Negative pools” had only SARS-CoV-2 negative samples (previously tested).

**Table 4:** Determination of sensitivity and specificity of SARS-CoV-2 detection on saliva pools (10 samples).

Pool samples	N	Positive	Negative	Sensitivity (95% CI)	Specificity (95% CI)
Positive	32	31	1	96.9% (0.84–0.99)	99.9% (0.87–1.00)
Negative	32	0	32		

Positive pools were constituted by 9 negative samples and 1 SARS-CoV-2 RNA spiked sample. The negative pools were constituted by 10 negative samples. A total of 260 saliva samples were randomly distributed in 32 pools of 10 samples. Sensitivity and specificity were determined as described [34–36]. CI–confidence interval.

**Table 5:** Determination of sensitivity and specificity of SARS-CoV-2 detection on saliva pools (20 samples).

Pool samples	N	Positive	Negative	Sensitivity (95% CI)	Specificity (95% CI)
Positive	32	28	4	87.5% (0.71–0.96)	99.9% (0.87–1.00)
Negative	32	0	32		

Positive pools were constituted by 19 negative samples and one positive SARS-CoV-2 RNA spiked sample. The negative pools were constituted by 20 samples. A total of 260 saliva samples were randomly distributed in 32 pools of 20 samples. Sensitivity and specificity were determined as described [34–36]. CI–confidence interval.

From the 32 “positive pools”, each formed by 10 saliva samples (9 negative and 1 positive; Table 4), 31 pools were detected as “positive” (average C_T_ of three genes: ORF1ab gene C_T_ 26.7; Envelope gene C_T_ 27.2 and Nucleocapsid gene C_T_ 26.6) and 1 as “not detected”. All 32 “negative pools” were detected as negative. Sensibility was determined to be 96.9% (95% CI: 0.84–0.99) and specificity was 99.9% (95% CI: 0.87–1.00). An almost perfect agreement was observed (Ƙ = 0.97, CI 95%: 0.91–1.00).

We also tested 20-sample pools (n = 64; Table 5). From the 32 “positive pools” (pools containing 1 control positive sample), 28 were detected as positive (average C_T_ of three genes: ORF1ab gene C_T_ 27.5; Envelope gene C_T_ 28.1 and Nucleocapsid gene C_T_ 27.0) and 4 were reported as “not detected”. All “negative pools” (n = 32) were detected as negative. Sensibility is 87.5% (95% CI: 0.71–0.96). Specificity is 99.9% (95% CI: 0.87–1.00). An almost perfect agreement was observed (Ƙ = 0.88, CI 95%: 0.78–0.96).

**Fig 3:**
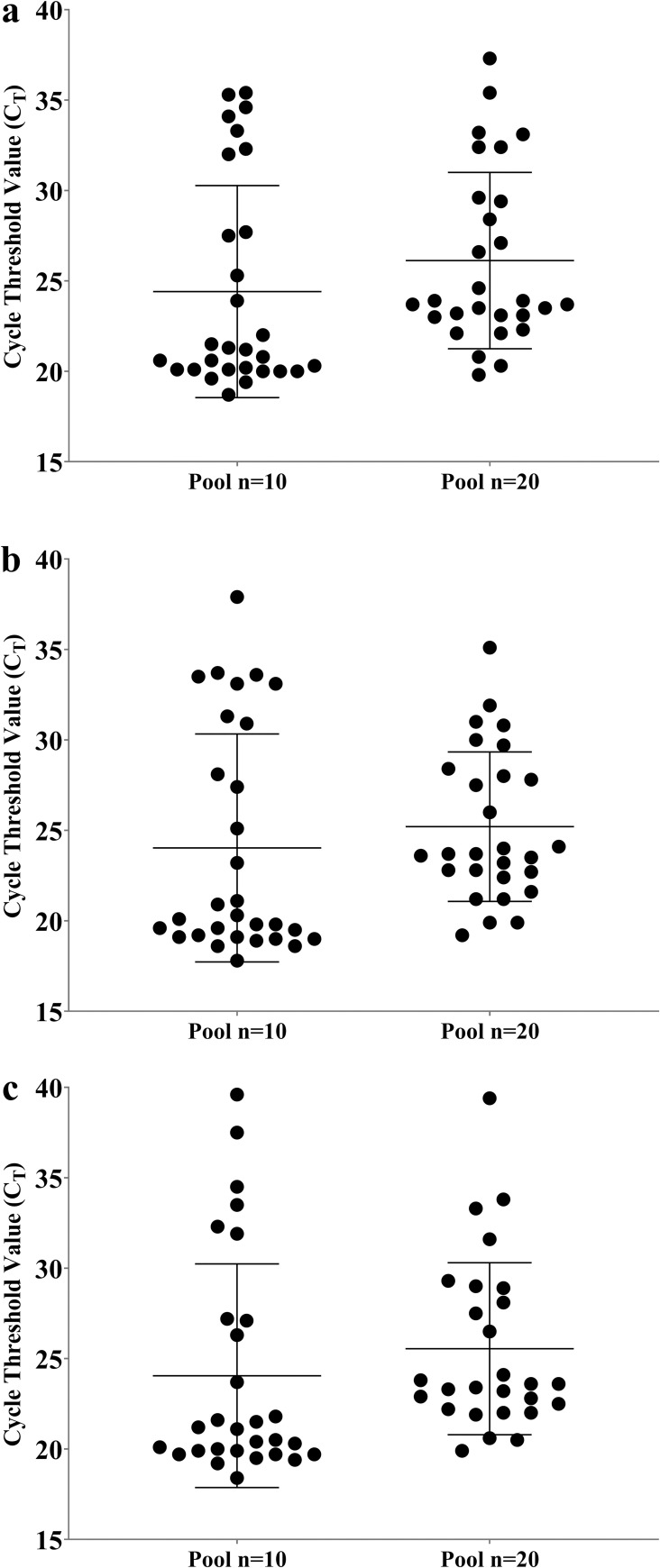
Comparison of Cycle Threshold (CT) values of 10- and 20-sample pools. CT values of Envelope (a), Nucleocapsid (b), and ORF1ab (c) genes were compared between the two types of samples by Mann-Whitney rank test.

The comparison of the Cycle Threshold value ORF1ab, Envelope and Nucleocapsid genes between 10-sample and 20-sample pools is shown on Fig 3. The 10-sample pool (20.93) showed slightly, non-significantly, lower C_T_ genes mean comparing to pool of 20 (23.70).

## Discussion

### Saliva assay sensitivity and reproducibility

The performance characteristics of COVID-19 PCR on saliva and other less invasive sample types, including throat gargle (oral rinses or mouthwashes), has only recently been evaluated [14, 37]. RNA extraction from saliva samples for SARS-CoV-2detection and viral load analysis is usually subjected to expensive, time consuming, kit-dependent protocols. We applied the SalivaDirect [33] methodology for saliva treatment. This methodology does not require expensive saliva collection tubes containing preservatives nor does it require specialized reagents or equipment for nucleic acid extraction. This strategy granted the reliable analysis of a high number of samples at low cost (Fig 1).

To assess the quality of detection of SARS-CoV-2 in saliva samples we compared the sensitivity and reproducibility of this method to the gold standard (nasopharyngeal samples). Previous studies showed that sensitivity for detection of SARS-CoV-2 RNA in saliva samples compared with NPS ranges from around 70% to 100% [8, 12, 15, 16, 27, 37, 38] and our results are in agreement with these studies. Besides being self-collected, saliva is less prone to errors than nasopharyngeal swab. False negatives have been related to swab collection technique, due to the heterogenicity on the deep insertion, and to the anatomy of the patient, introducing a higher level of variability [1, 2, 27, 37].

Viral loads considering C_T_ values are not only the threshold used for considering a test result positive but also frequently used as a comparison measure between test results. In the current study, the overall median C_T_ in SAL was slightly higher than NPS, although the difference was not statistically significant (*p*>0.05). Silva et al [39] showed that nasopharyngeal and saliva viral load, are not equivalent measures of disease processes for COVID-19. The correlation between nasopharyngeal and saliva was low (R = 0.61) and while saliva viral load could significantly predict disease severity and mortality over age, nasopharyngeal viral load could not reliably distinguish severity or predict mortality [40]. So far there is no consensus as to which of the sample types, SAL or NPS, has higher viral load values. Some studies have reported that higher viral loads were seen in patients with more severe disease. In our study, the C_T_ values are not statistically different in SAL (reflecting a lower viral load) from NPS samples. Importantly, the range of viral load in the specimens in a lower number of samples can greatly affect the final calculated percent positive agreement because the specimens with higher viral loads are more likely to be detected by both NPS and SAL; therefore, studies with a higher median viral load across most specimens will show a higher percent positive agreement than a study with a lower median viral load [27].

### Pooling saliva samples for SARS-CoV-2 screening

To date, only a few studies have evaluated the efficiency of SARS-CoV-2 detection in pooled saliva samples [13, 27–29]. Some studies analysed the efficiency in different pooling sizes from 5 [27, 29], 10 [13, 27] to 20 [28] samples. If, on the one hand, pools with a smaller number of samples suggest greater sensitivity in detecting the virus, on the other hand, the cost reduction increases considerably with the increase in pool size. The ideal is to be able to increase the size of the pools without losing the ability to detect the virus. This is especially relevant in individuals with a low viral load. In our study we tested pools of 10 and 20 saliva samples applying the methodology to real situations.

We showed that SARS-CoV-2 can be detected both in 10 and 20-sample saliva pools, although the sensitivity is 9.4% lower in the latter. The lower sensitivity on 20-sample pools is surely associated to the dilution factor. Previous studies also demonstrated an increase in Ct values after pooling [27–29]. Not all studies used direct sample pooling, and some used extracted RNA to build the pools, with increase resource use (time and cost) and is prone to error. Pasomsub et al 2020 [29] used extracted RNA pooling strategy to build pools of 5 and 10 samples, achieving lower differences (0.1 and 1.4 respectively) in the C_T_ values of individual samples and pool. These C_T_ values are lower than saliva sample pooling. On other studies, an average increase 2–3 C_T_ was obtained on 5- and 10-sample pools, and an increase of almost 4 C_T_, on 20-sample pools [13, 27, 29]. Our saliva pooling strategy, resulted in only a slight increase on C_T_ value of 1.2 C_T_ (in 10 sample pools), and 1.9 C_T_ in average (in 20 sample pools) compared to positive spike samples.

Recently some reports described saliva as an appropriate fluid for SARS-CoV-2 detection on patients with high viral load [27]. Nonetheless, we were able to detect infected, but asymptomatic patients. One advantage of pool testing is its time and cost-effectiveness, allowing population-based asymptomatic screening or monitoring even when testing supplies are limited.

The pooling strategy described herein was applied to SARS-CoV-2 testing in a real-life scenario. Over 2000 individuals were tested with this strategy being possible to identify 3 asymptomatic SARS-CoV-2 carriers (0.15%), preventing outbreaks. The application of this strategy to a population of civil servants and junior athletes provided the economy of 1840 individual tests. Also, pools were built with saliva samples instead of extracted viral RNA. We demonstrated the efficiency of saliva pooling for the detection of SARS-CoV-2 heterogeneous population under investigation for COVID-19 in a low prevalence setting. Saliva pooling may facilitate the detection of the disease in suspected symptomatic patients during the disease outbreak, providing the advantages in the ease of specimen collection and resource conservation.

Nevertheless, some aspects of this study deserve attention. First, although almost 300 samples were used, the declining incidence of COVID-19 cases over the study period limited the number of positive samples enrolled in the study. Although saliva collection is easier and less prone to variation than nasopharyngeal collection, saliva can be a challenging sample when it comes to processing and analysis. Therefore, rigorous methods of homogenization and pipetting of samples must be followed. Furthermore, it is important that when using saliva samples, the C_T_ used for considering a sample positive should be adjusted. Most detection kits are optimized for nasopharyngeal swabs, and as our and other studies suggest, C_T_ tend to be higher in saliva samples. In this study the C_T_ was increased from the recommended 32 cycles to 40 cycles guaranteeing the high sensibility and specificity of the SARS-CoV-2 detection.

## Conclusion

In summary, we have demonstrated that saliva samples can be reliably used for SARS-CoV-2 detection, and that saliva-based large-scale population screening for COVID-19 with or without pooling is feasible, fast and leads to large economic and environmental impact reductions. We showed that saliva pools of either ten or twenty samples do not compromise the detection of SARS-CoV-2. The ease of saliva collection, and the pool strategy is an appealing method for mass-screening programs or sentinel surveillance, especially in resource-limited scenarios.

## Supporting information

S1 Table Population data from community testing with pooled sample analysis strategy. (XLSX)
